# P-896. Artificial Intelligence Performs Well in Assessing the Clarity of Definitions of Variables in Periprosthetic Joint Infection Studies

**DOI:** 10.1093/ofid/ofae631.1087

**Published:** 2025-01-29

**Authors:** Bettina Gabrielle V Tenorio, Melody Hope Lee Yu, Angelica Bernadette Deslate, Don Bambino Geno Tai

**Affiliations:** Ateneo School of Medicine and Public Health; NYC Health + Hospitals / Elmhurst, Icahn School of Medicine at Mount Sinai, Woodside, New York; Ateneo de Manila University, Manila, National Capital Region, Philippines; Ateneo de Manila University, Manila, National Capital Region, Philippines; University of Minnesota, Minneapolis, Minnesota

## Abstract

**Background:**

Artificial intelligence (AI) has the potential to improve healthcare research methodologies, particularly in addressing the need for consistency and clarity in defining variables in observational studies. However, concerns exist regarding the accuracy and reliability of AI in this context.

Performance of Artificial Intelligence in Assessing Clarity of Definition of Variables

**Figure:**
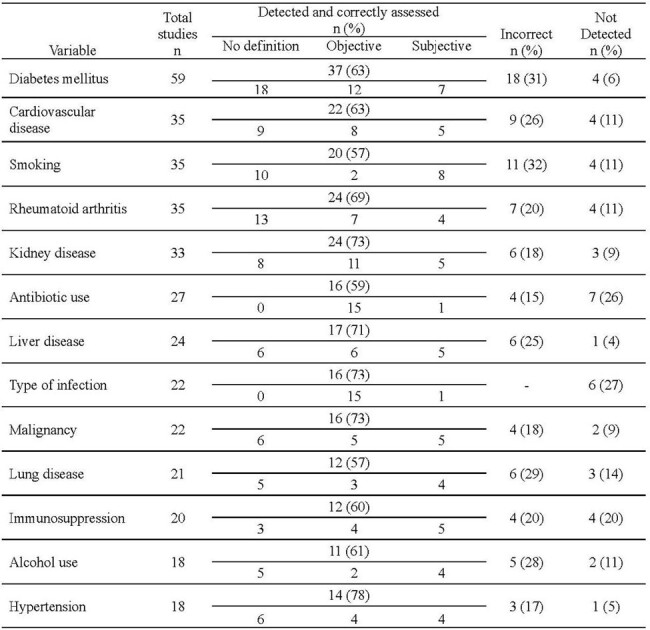

**Methods:**

We reviewed observational studies on prosthetic joint infection studies from 2017-2023, focusing on 13 categories of variables. We used ClaudeAI 2 (Anthropic, San Francisco, CA), a large language model, in analyzing definitions from full texts, instructing it to classify them as objective, subjective, or undefined. Some examples of a highly objective definition are the presence of time element, severity, staging, frequencies, laboratory cut-off, and medication dependence. A simple chart review was deemed subjective.

**Results:**

We included 75 studies with a total of 369 variables in the analysis. AI detected 88% of variables (n=324). Of those detected, 74% (241/324) were correctly classified. It accurately identified antibiotic use and infection type as the variables that are most frequently defined objectively (94%). For the 9/13 categories, it appropriately assessed that most studies lacked definitions. Smoking (20%) and alcohol use (33%) were least frequently defined. Notably, 5 detected variables did not exist in the manuscripts.

**Conclusion:**

The model demonstrated high performance in detecting variables from study text and evaluating the clarity of variable definitions. The false positive detections serve as an important reminder that human oversight remains crucial to ensuring accuracy when integrating AI into the research process. Looking ahead, AI may assist researchers in objectively defining study variables from the outset and verifying the correct application of definitions. More studies are needed on how to optimally integrate its capabilities into the research process.

**Disclosures:**

**All Authors**: No reported disclosures

